# EMANCIPATORY GERONTOLOGY AND THE POLITICAL ECONOMY OF THE CITY: NEW INEQUALITIES AND POLICY RESPONSES

**DOI:** 10.1093/geroni/igae098.0178

**Published:** 2024-12-31

**Authors:** Chris Phillipson, Tine Buffel

**Affiliations:** University of Manchester, Manchester, England, United Kingdom; The University of Manchester, Manchester, England, United Kingdom

## Abstract

Carroll Estes identifies a central aim of an ‘emancipatory gerontology’ as ‘advancing knowledge and the realization of dignity, access, equity and social justice through individual and collective agency and social institutions.’ Wright argues that the word ‘emancipation’ signals a central moral purpose in the production of knowledge, with ‘the elimination of oppression and the creation of conditions for human flourishing’. But the achievement of these aims has been undermined with the dismantling of the welfare state, extremes of economic inequality, and the expansion of precarious employment. The impact of such trends on groups such as older people continue to be the subject of extensive research. However, of additional concern are divisions created by economic and social forces operating within urban environments. Savage argues that large cities are not just products but drivers of inequality, with many European and North American cities no longer serving people from a wide range of communities, reflected in the decline of affordable housing, and the impact of gentrification. The presentation will consider how a political economy of the city, drawing on research in urban sociology, can re-vitalise a critical gerontology, bringing new perspectives on ageing and the life course. The paper will explore how this approach can be used as a framework for understanding new forms of inequality affecting later life. It will conclude by arguing that different ideas are needed for ‘thinking about’ ageing within cities, highlighting how innovations produced by urban environments can serve as a catalyst for emancipation and liberation from oppression.

